# Study of Changes in Crystallinity and Functional Properties of Modified Sago Starch (*Metroxylon* sp.) Using Physical and Chemical Treatment

**DOI:** 10.3390/polym14224845

**Published:** 2022-11-10

**Authors:** Herlina Marta, Hana Nur Layalia Hasya, Zahra Indah Lestari, Yana Cahyana, Heni Radiani Arifin, Siti Nurhasanah

**Affiliations:** 1 Department of Food Technology, Faculty of Agro-Industrial Technology, Universitas Padjadjaran, Bandung 45363, Indonesia; 2 Research Collaboration Center for Biomass and Biorefinery between BRIN and Universitas Padjadjaran, Bandung 45363, Indonesia

**Keywords:** crystallinity, functional properties, modification, sago starch

## Abstract

Sago starch has weaknesses such as low thermal stability and high syneresis. Modifications were made to improve the characteristics of native sago starch. In this study, sago starch was modified by autoclave-heating treatment (AHT), osmotic-pressure treatment (OPT), octenyl-succinic anhydride modification (OSA), and citric acid cross-linking (CA). This study aimed to examine the changes in chemical composition, crystallinity, and functional properties of the native sago starch after physical and chemical modifications. The results show that physical modification caused greater granule damage than chemical modification. All modification treatments did not alter the type of crystallinity but decreased the relative crystallinity of native starch. New functional groups were formed in chemically modified starches at a wavelength of 1700–1725 cm^−1^. The degree of order (DO) and degree of double helix (DD) of the modified starches were also not significantly different from the native sample, except for AHT and OPT, respectively. Physical modification decreased the swelling volume, while chemical modification increased its value and is inversely proportional to solubility. AHT and OPT starches have the best freeze–thaw stability among others, indicating that both starches have the potential to be applied in frozen food.

## 1. Introduction

Sago is one of the potential local commodities originating from the eastern part, which is abundant in Indonesia. This commodity has a high potential as an alternative to local starch because it has high starch productivity, which is more than 400 kg of dry starch in each sago tree trunk. Sago flour production reaches 25 MT/ha, several times more than potato or cassava flour [1]. The Directorate General of Plantations from the Ministry of Agriculture stated that sago production in Indonesia would increase in 2021 to 381,965 tons, with Riau province as the largest producer, with 275,807 tons. In contrast to the abundant production, the sago consumption level showed a relatively low number. According to the Ministry of Agriculture of Indonesia, data on sago consumption show a low number, that was 0.4–0.5 kg/capita/year. The availability of large quantities of sago in Indonesia should be maximized as well as possible, considering that sago starch is one of the local food ingredients that has potential as a functional food because it has a low glycemic index, high amylose, and valuable antioxidant content.

The native starch produced has some limitations in its physical, chemical, and functional properties, such as being unstable to mechanical treatment, easy to retrograde, hard, sticky, and unclear pasta formed, and low thermal and acid stability. Furthermore, Nordin et al. [2] have reported that native sago starch has weaknesses such as instability in mechanical treatment, high temperature, and high pH. Wild sago starch is also easily retrograded and has high syneresis.

Several modifications were performed to improve the weaknesses of sago starch. In this study, physical and chemical modifications of starch were carried out. Several methods in physical modification are commonly involved hydrothermal treatment or pre-gelatinization processes to improve the native starch properties, such as annealing, heat-moisture treatment, pre-gelatinization, etc. Heat-moisture treatment (HMT) has been widely used and produces starch characteristics that are more resistant to heat and mechanical processes. HMT is a hydrothermal treatment, where the starch is treated at high temperatures (80–120 °C) and low moisture levels (less than 30%) for a certain period [3,4]. However, in this study, HMT was carried out using the autoclave-heating treatment (AHT) and osmotic-pressure treatment (OPT), which involved high temperature and pressure from the autoclave. The difference between AHT and OPT is the presence of saturated sodium sulfate as a reagent in OPT, which can prevent starch gelatinization [5].

The chemical modification consists of several methods, including cross-linking, esterification, etherification, oxidation, etc. In contrast to physical modification, chemical modification involves the introduction of functional groups into the starch molecule, resulting in changed physicochemical and functional properties [6]. In this study, the chemical modifications have been carried out using octenyl-succinic anhydride (OSA) and citric acid as a reagent. The OSA method is a modification of starch esterification with octenyl functional groups, performed by partial substitution, and the hydroxyl group of starch is changed to hydrophobic so that the starch is amphiphilic [7]. Chemical modification using OSA has been studied previously to improve native starch characteristics [8]. The OSA starch can act as an emulsifier and encapsulating agent in food products [9]. Whereas modification using citric acid is one of the esterification methods in which a cross-linking process occurs. Citric acid reacts with many hydroxyl groups in starch to produce products with a high degree of substitution [10]. In addition, this modification can convert hydrophilic hydroxyl groups into hydrophobic ones and prevent retrogradation during storage [11]. The study comparing the effect of physical and chemical modifications on sago starch characteristics was still limited, so in this study, the alteration of the granule morphology, crystallinity, and functional properties of sago starch caused by both modification methods was examined. This information can be used to expand the application of sago starch in the food industry.

## 2. Materials and Methods

### 2.1. Materials

The raw material used in this research is sago starch (*Metroxylon* sp.) (Javara, Jakarta, Indonesia). All chemicals used for modification, such as 2-octen-1-ylsuccinic anhydride (OSA) (Sigma-Aldrich, St. Louis, MO, USA), acetic acid, hydrochloric acid, and sodium hydroxide, were of analytical grade.

### 2.2. Autoclave Heating-Treated (AHT) Starch Preparation

The autoclave heating-treated starch was prepared using the previous method reported by Marta et al. [12]. The starch sample was adjusted to 20% moisture content. The moistened starch was then equilibrated in a refrigerator at 4 °C for 24 h and stored in glass bottles, and then heated using an autoclave at 120 °C for 1 h. AHT starch was removed from the Duran bottle after cooling to room temperature and dried at 50 °C for 24 h in a drying oven (Shel Lab FX-14-2). Then, it was milled and sieved to a particle size of ≥100-mesh.

### 2.3. Osmotic Pressure-Treated (OPT) Starch Preparation

The osmotic pressure-treated starch was prepared using the previous method [12]. Starch was dissolved in a saturated sodium sulfate solution. The saturated sodium sulfate solution was prepared by dissolving 100 g of Na_2_SO_4_ per 200 mL of distilled water. The starch suspension made in glass bottles was heated using an autoclave, and the device was set at a temperature of 120 °C for 1 h. The heated sample was then cooled to room temperature. The sample mixture was washed using 500 mL of distilled water 8 times using centrifugation at 5000 rpm for 3 min. The obtained starch was dried at 50 °C for 24 h in a drying oven (Shel Lab FX-14-2). Then, it was milled and sieved to a particle size of ≥100-mesh.

### 2.4. Octenyl-Succinate Anhydride (OSA) Starch Preparation

The OSA modification method refers to the method by Anwar et al. [13]. In this study, the sample was dissolved with distilled water, and 1 M NaOH was added until the pH rose to 8.0. Then, OSA was slowly dripped into the starch solution to maintain the pH at 8.0–8.5. When the pH was stable, it was stirred for 3 h, and then the pH was lowered to 6.5 using HCl. The sample was washed thrice at a speed of 5000 rpm for 3 min. Then, the washed samples were placed into a tray and then dried in a blower oven for 24 h at 50 °C. The dry sample was then milled and sieved to a particle size of ≥100-mesh.

### 2.5. Citric Acid Cross-Linked (CA) Starch Preparation

The citric acid cross-linked starch method refers to a previous study [14], with a slight modification. The sago starch was then added with a solution of citric acid made of 27 g of citric acid and 101.53 g of distilled water until the pH changed to 3.5. After that, the samples were stored at room temperature for 16 h and then stored in the oven cabinet for 24 h. After the drying process, the samples were roasted at 130 °C for 5 h. The samples were then washed three times at a speed of 5000 rpm for 3 min. Then, the washed samples were placed into a tray and then dried in a blower oven for 24 h at 50 °C. The dry samples were then milled and sieved to a particle size of ≥100-mesh.

### 2.6. Chemical Composition and Amylose Content Determination

Moisture content was determined by referring to the AOAC [15] method, which was then gravimetrically determined. Starch and amylose contents were determined by Luff-schoorl [16] and the direct acid hydrolysis method [17], respectively.

### 2.7. Granular Morphology Determination

Granular morphology was determined using scanning electron microscopy (SEM) (JEOL, Akishima City, Japan, JSM-6360 LA at 15 kV) [18]. Starch samples were spread on an aluminum plate and coated with gold/palladium at 8–10 mA for 10–15 min with pressure below 10 torrs. Representative digital images of starch granules were obtained at 1000× magnification.

### 2.8. Particle Size Analysis

The particle size distribution of starch was determined using a Particle Size Analyzer (PSA) (LS 13 320 MW, Backman Coulter, Brea, CA, USA) [19]. Starch was suspended in distilled water and stirred until homogeneous. The refractive index (RI) of distilled water as dispersion was 1.33 and 1.52. The starch suspension that formed was injected into the cell samples. Measurements were carried out until a stable value was reached.

### 2.9. Starch Crystallinity

The crystallinity of sago starch samples was analyzed using PANalytical, Malvern, UK, X’Pert PRO series PW3040/x0. Before analysis, the moisture content of samples was adjusted to 6% ± 0.5%. This instrument was used to collect the X-ray diffraction pattern with a copper tube operating at 40 kV and 30 mA. This diffractometer produces the Cu-K α-radiation with the wavelength of 1.5406 Å. The diffractogram of the starch samples was obtained by scanning the sample from 5° to 50° (2θ) at room temperature, with an increase of 0.02° every second, with a rotating speed of the holder of 30 min^−1^. Relative crystallinity was determined using a sigma plot program by calculating the area under the curve of the crystalline region and the amorphous region in the obtained diffractogram [19]. Relative crystallinity (RC) was calculated using Equation (1):

(1) $$\mathrm{RC}=\;\frac{\mathrm{Crystalline}\;\mathrm{area}}{\mathrm{Crystalline}\;\mathrm{area}\;+\;\mathrm{Amorphous}\;\mathrm{area}}\times \;100\%$$

### 2.10. Functional Groups and Molecular Order Degree (FTIR-ATR)

FTIR was used to analyze functional groups of sago starch samples. Samples were mixed with KBr (1:3 *w*/*w*) and pressed into pellets and then subjected to attenuated total reflectance (ATR). The wavelength range and spectral resolution used were 4000–500 cm^−1^ and 8 cm^−1^ by 32 scans, respectively. Degree of order (DO) and degree of the double helix (DD) were also measured by the absorbance ratios of 1047/1022 cm^−1^ and 995/1022 cm^−1^, respectively [18].

### 2.11. Swelling Volume and Solubility

Swelling volume and solubility were measured using the method by Marta et al. [20]. Sago starch samples (0.35 g) were placed in a tube and mixed with distilled water (12.5 mL) and then heated in a water bath at 92.5 °C and stirred regularly for 20 min. The starch sample was cooled for 1 min in ice water and centrifuged at 3500 rpm for 30 min. The supernatant was separated, measured, and dried in the oven to calculate the solubility. The swelling volume and solubility were calculated using Equations (2) and (3), respectively:


(2) $$\mathrm{Swelling}\;\mathrm{volume}\;\left(\mathrm{mL}/g\right)=\frac{\mathrm{total}\;\mathrm{volume}-\mathrm{swelling}\;\mathrm{volume}}{\mathrm{weight}\;\mathrm{of}\;\mathrm{sample}\;\left(\mathrm{db}\right)}$$

(3) $$\mathrm{Solubility}\;\left(\%\right)=\frac{\mathrm{weight}\;\mathrm{of}\;\mathrm{dry}\;\mathrm{supernatant}}{\mathrm{weight}\;\mathrm{of}\;\mathrm{sample}\;\left(\mathrm{db}\right)}\times 100$$

### 2.12. Water Absorption Capacity (WAC)

Water absorption capacity was measured using the method by Marta et al. [19]. Distilled water (10 mL) was added to sago starch samples (1 g) in a centrifuge tube, and vortexed. The solutions were then conditioned at 26 ± 2 °C for 1 h and centrifuged at 3500 rpm for 30 min. The volume of supernatant was measured, and WAC was calculated using Equation (4):


(4) $$\mathrm{WAC}\;\left(g/g\right)=\frac{\mathrm{volume}\;\mathrm{of}\;\mathrm{water}\;\mathrm{absorbed}}{\mathrm{weight}\;\mathrm{sampel}\;\left(\mathrm{db}\right)}$$

### 2.13. Oil Absorption Capacity (OAC)

Oil absorption capacity was measured using the method by Dewi et al. [21]. First, 10 mL of vegetable oil was added to 1 g of sago starch samples in a centrifuge tube and vortexed. The solutions were then conditioned at 26 ± 2 °C for 1 h and centrifuged at 3500 rpm for 30 min. The volume of supernatant was measured, and OAC was calculated using Equation (5):


(5) $$\mathrm{OAC}\;\left(g/g\right)=\frac{\mathrm{volume}\;\mathrm{of}\;\mathrm{oil}\;\mathrm{absorbed}}{\mathrm{weight}\;\mathrm{sampel}\;\left(\mathrm{db}\right)}$$

### 2.14. Freeze–Thaw Stability (% Syneresis)

Freeze–thaw stability was measured using the method of Wattanachant et al. [22]. Sago starch suspension was made with a solid concentration of 5% and then heated at 95 °C for 30 min with constant stirring for complete gelatinization. Starch paste (20 g) was put into a centrifuge tube and stored at 4 °C for 24 h before freezing at −20 °C for 48 h. The frozen samples were then thawed at room temperature for 2–3 h and centrifuged at 3500 rpm for 15 min. The supernatant was then weighed and the percentage of syneresis was calculated by dividing the weight of the supernatant by the weight of the starch paste (Equation (6)):


(6) $$\mathrm{Syneresis}\;\left(\%\right)=\frac{\mathrm{weight}\;\mathrm{of}\;\mathrm{supernatant}}{\mathrm{weight}\;\mathrm{of}\;\mathrm{total}\;\mathrm{sample}}\times 100$$

### 2.15. Statistical Analysis

Data are presented as the mean ± SD of triplicate experiments. All data were analyzed by one-way analysis of variance (ANOVA) with a further Duncan’s test to compare the sample mean at a significance level of 5% (*p* < 0.05). All data were analyzed using Statistical Software Program (SPSS) version 26.0.

## 3. Results

### 3.1. Chemical Composition

The chemical composition of native and modified sago starch observed was moisture, starch, and amylose content (Table 1). The moisture content of sago starch was significantly different between native and modified sago starch. Both physical and chemical modifications had a similar effect on native sago starch, which was reducing its moisture content. The starch content of each modified starch ranged from 85% to 87%, which indicates that the non-starch component in sago starch was 13–15%. Starch content between autoclave-heating treatment (AHT), osmotic-pressure treatment (OPT), and cross-linking citric acid (CA) starches was not significantly different, but it significantly increased in octenyl-succinic anhydride (OSA)-modified starch. The increase in starch content has also been reported by another study [23]. This was due to the esterification process, which can increase the molecular weight of starch. Amylose content of all modified sago starches was not significantly different from native starch, except for AHT starch. Amylose content of native starch increased after heat-moisture treatment (HMT), which might be due to the breaking of the amylopectin chain, which turned into an amylose linear chain [22].

### 3.2. Granule Morphology

**Figure 1:**
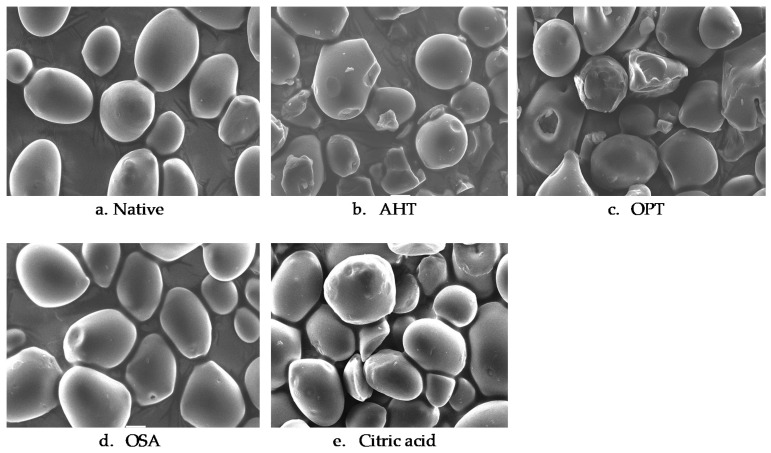
Granule morphology of native and various modified sago starches (1000×). AHT = autoclave-heating treatment, OPT = osmotic-pressure treatment, OSA = octenyl-succinic anhydride, CA = citric acid cross-linking.

Granule morphology of native and modified sago starch was observed by scanning electron microscope (SEM), and corresponding images are presented in Figure 1. The native starch granules had a slightly oval shape and smooth surface without pores, which was in line with a previous study [24]. Physically modified starches, both AHT and OPT, showed more severe granule damage than chemically modified starches. The use of heat with limited moisture content in physical modifications caused the granules’ surfaces to become rough, cracked, and some were broken. While chemically modified starches, both OSA and CA starches, did not show any extreme change on the granule surface, some granules’ surfaces had indentation, but most of the granules retained their original shape and smooth surface.

AHT starch showed an irregular-shaped granule surface, cracks, and a rough surface. Most of the granule particles had an incomplete shape, and were broken and fragmented, which might be due to the partial gelatinization of AHT starch granules caused by the presence of limited water and heat energy in the starch system [18]. Similar findings were reported in hydrothermally modified breadfruit and banana starch [3,12]. OPT starch produced granule morphology with an irregular shape that had cracks, and some parts of the granules had an indentation. The presence of sodium sulfate solution can pressure the granules during the heating process [12]. The osmotic pressure generated in the solution system causes the plasmolysis of starch granules [18]. Similar results were reported in OPT-modified potato starch and taro starch [5,25].

Chemical modification of sago starch did not significantly change the shape of the granule surface compared to native starch. OSA starch mostly had a smooth texture, and there was minor damage in a small portion of the starch granules. Small amounts of the granules showed small holes and cavities and a slight depression at the ends of the granules due to the esterification treatment. This small change was triggered by the esterification reaction and hydrolysis due to the addition of NaOH at the beginning of the treatment [26]. OSA can only react on the surface of the starch granules because OSA has low solubility in water [27]. Similar findings were reported in waxy corn and potato starch [28,29]. Several granule surfaces of CA starch had little cracks and small agglomerations caused by the addition of citric acid. Remya et al. [23] reported that after the cross-linking modification process using citric acid, the granule structure of cassava starch and potato starch did not cause a significant change in granule morphology.

### 3.3. Particle Size Distribution

**Figure 2:**
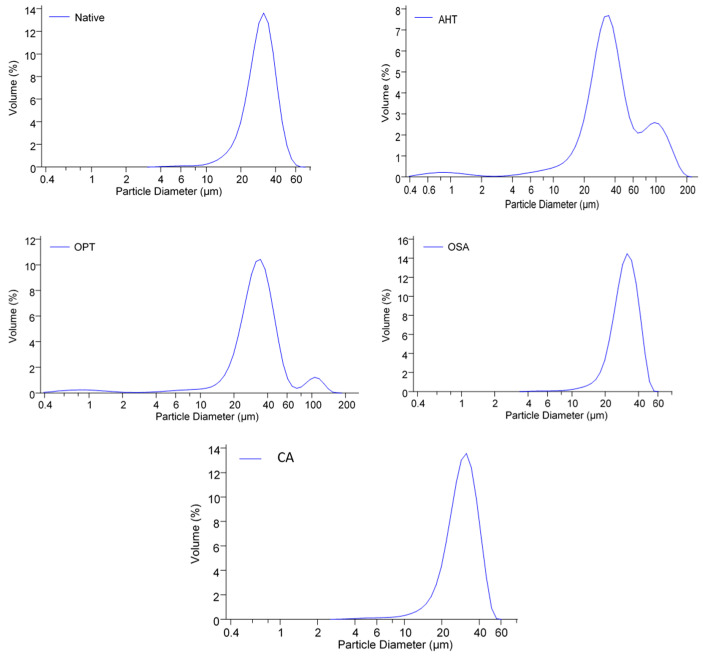
Particle size distribution of native and various modified sago starches. AHT = autoclave- heating treatment, OPT = osmotic-pressure treatment, OSA = octenyl-succinic anhydride, CA = citric acid cross-linking.

Figure 2 shows that native starch had a size distribution curve of one peak (unimodal). The chemical modification did not alter the size distribution curve of native starch, whereas the physical modification altered it from unimodal to bimodal (two peaks).

AHT- and OPT-modified starch increased the granule size of native starch (Table 2). The increase in starch granule size in AHT was due to the entry of water into the starch granules during the initial conditioning of the starch moisture content. OPT starch also increased, but not as much as the increase in AHT starch due to the limitation of gelatinization due to the presence of sodium sulfate solution. Thermal treatment caused the breaking of hydrogen bonds, thus facilitating water absorption and undergoing gelatinization at high temperatures. OSA-modified starch had a higher starch granule size than native starch, which indicated the increasing number of hydrophobic groups [30]. The citric acid cross-link modification decreased the granule size, which was in line with a previous study [31]. Furthermore, Zhou et al. [31] reported that the cross-link modification significantly reduces the particle size and narrows the size distribution.

### 3.4. Starch Crystallinity

**Figure 3:**
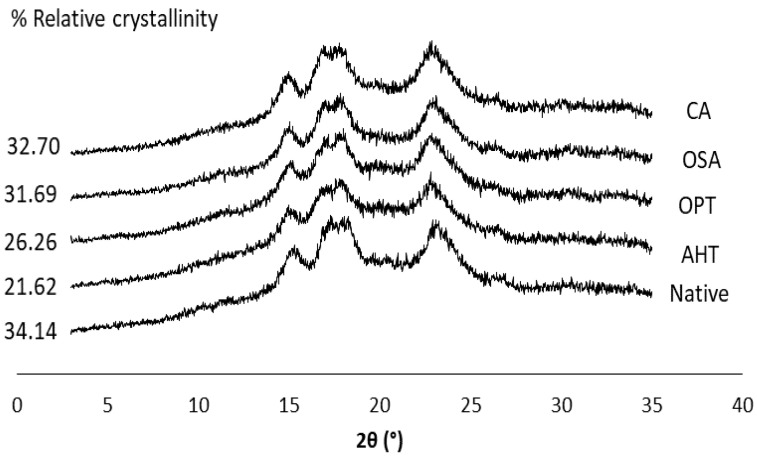
X-ray diffraction patterns of native and various modified sago starches. AHT = autoclave-heating treatment, OPT = osmotic-pressure treatment, OSA = octenyl-succinic anhydride, CA = citric acid cross-linking.

The native sago starch has an A-type crystallinity pattern with firm peaks at 15° and 23° and unresolved peaks at 17° and 18°. All modification methods did not alter the crystallinity pattern of native sago starch (Figure 3).

Although it did not change the type of crystallinity pattern, the relative crystallinity (RC) of all modified sago starches decreased compared to the native starch. The RC of physically modified sago starch tends to be lower than that of chemical modification. Following a study by Marta et al. [12], the RC of native breadfruit starch decreased after thermal modifications such as AHT and OPT, which was due to the heating treatment at high temperatures, which destroys the crystalline structure [14]. Furthermore, Zavareze and Dias [4] have reported that hydrothermal treatment can also increase the amorphous area in semi-crystalline lamellae so that the crystallinity area was reduced. Chemically modified starch has a greater RC than physically modified starch, but lower than native starch. The tendency to decrease crystallinity in chemically modified starch has also been reported by No et al. [32] for OSA-modified Japonica rice starch. Esterification of starch with OSA mainly occurred in the amorphous area, and might affect the crystalline area, resulting in a decrease in relative crystallinity, which was in line with another study [33]. Furthermore, Whitney et al. [34] reported that OSA substituted on starch molecules depending on the presence of pores in starch granules and the OSA level. At the 3% OSA level, OSA substituted mainly on amylose chains or possibly on amylopectin chains that have been hydrolyzed from the amylopectin molecules during esterification.

### 3.5. Molecular Degree Order

**Figure 4:**
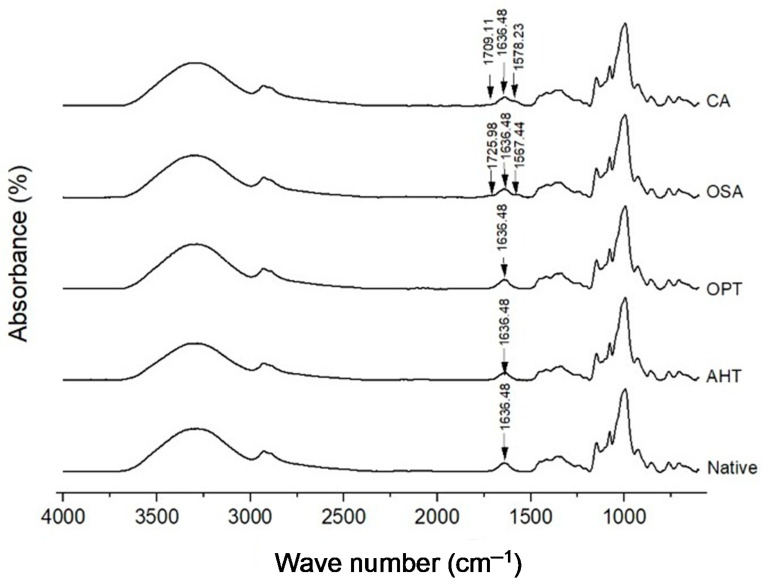
FTIR spectra of native and various modified sago starches. AHT = autoclave-heating treatment, OPT = osmotic-pressure treatment, OSA = octenyl-succinic anhydride, CA = citric-acid cross-linking.

FTIR spectra of native and modified sago starches are shown in Figure 4. All samples have the same peak at a wavelength of 3000–3700, indicating a hydrogen-bonded hydroxyl group in the starch structure (-OH) [35]. Subsequent small peaks formed at wavelengths of 2850–2950, which indicated C-H bonds, then small and sharp peaks formed again around 1636.48, which indicated the interaction of water with starch granules, then formed several small peaks at wavelengths of 1200–1500, which represented C-C, C-O, and C-H strains. The highest and sharpest peak was at wavelength 1000, which was the general characteristic of polysaccharides [36]. OSA and CA modifications changed the molecular structure of native starch. This can be seen through the presence of two new functional groups formed around waves at 1725.98 and 1567.44 for OSA-modified sago starch. These results were in line with previous studies [37,38]. The new functional group at 1725 cm^−1^ indicated the formation of an ester carbonyl group in the starch [38]. The formation of new functional groups was also seen in the modification of citric acid cross-linking, where the functional groups were formed at wavelengths of 1709.11 and 1578.23. This newly formed peak can be attributed to the stretching of the C=O vibration but has a low intensity because the ester bond content is relatively low in cross-linking [39]. The formation of new functional groups in modified starch by cross-linking citric acid has also been reported in a previous study [40].

From FTIR, we could also measure the degree of order and internal changes in the degree of the double helix of starch granules, which was calculated by the ratio of the absorbance band at 1047 and 1022 cm^−1^ and the ratio of the absorbance band at 995 and 1022 cm^−1^, respectively. Table 3 shows that the physical and chemical modification treatments did not significantly affect the degree of order (DO) of native sago starch, except for AHT starch. AHT starch had a lower DO than its native counterpart. It was supported by another study by Marta et al. [18] that decreased DO in AHT is due to the high heat and pressure involvement, causing more significant structural damage. Although both are physical modifications, the DO values of AHT- and OPT-modified starch were significantly different. It might be due to the sulfate component, which can minimize the damage and inhibit gelatinization in OPT starch. The chemical modifications such as OSA and CA did not significantly change the DO value because they did not involve high heat treatment, so it had a better level of regularity.

The degree of the double helix (DD) of all modified starches was not significantly different from native starch, except OPT starch. Increased DD in OPT was due to the presence of saturated sodium sulfate solution. Although the modification involves heat, the sulfate can prevent excessive gelatinization. Compared with both physical modification treatments, it was seen that DD between AHT and OPT was not significantly different. Similarly, between both chemical modification treatments, OSA and CA. Velásquez-Barreto et al. [41] have reported that the OSA modification decreased the DD value of starch isolated from *Oxalis tuberosa* (oca) tubers. In this study, the DD of OSA starch also tended to decrease, but statistically, it was not significantly different from the DD of native starch.

### 3.6. Functional Properties

#### 3.6.1. Swelling Volume and Solubility

Table 4 shows that all modification treatments significantly affected the swelling volume (SV) of native sago starch. The physical modification decreased the SV, whereas the chemical modification increased the SV. The decreased SV in physically modified starch was reported by some other researchers [12,42]. The thermal modification caused more crystal arrangements in polymer granules, which reduced water penetration and inhibited starch swelling. In addition, SO_4_^2−^ ions in the OPT modification can inhibit the absorption of water by starch granules. Furthermore, Bhosale and Singhal [43] have reported that there is a substitution of the acetyl group with the hydroxyl group in OSA starch molecules, which causes weakening of the hydrogen bond and the starch granule structure. This causes the granules to be more easily penetrated by water and makes it easier for them to swell.

The solubility (SOL) of all modified sago starches was significantly different from native sago starch (Table 4). SOL has a reverse trend with SV, whereby the physical modification increased the SOL whereas the chemical modification decreased the SOL of native sago starch. AHT starch had the highest SOL, which was significantly different from the other physical modification (OPT). The increase in SOL of AHT starch was due to an increase in the amorphous region, which was readily soluble in heat compared to the crystalline region [12]. The increase in SOL also occurred in OPT-modified starch, which was in line with another study on OPT corn starch [18] and on OPT breadfruit starch [12]. OPT can inhibit starch gelatinization and swelling [44]. Furthermore, Pukkahuta et al. [45] showed that a decrease in swelling of OPT was caused by a decrease in enthalpy of gelatinization, which increased the amorphous region of the starch granules. Furthermore, the amorphous region was susceptible when dissolved in hot distilled water, leading to an increase in SOL.

In contrast to physically modified starch, the SOL of OSA and CA starches decreased compared to their native counterpart. The decrease of SOL on CA starch was due to the higher molecular weight, making it difficult to dissolve [46]. Furthermore, Xiao et al. [47] have reported that the decrease in SOL of CA starch might be caused by the starch granule structure being strengthened by cross-linking. OSA starch had the lowest SOL compared to the other modifications. The decrease in SOL of OSA starch was due to the presence of a very strong bond between amylose and substituent groups so that water molecules were trapped in starch molecules and prevented amylose molecules from leaching out.

#### 3.6.2. Water Absorption Capacity (WAC), Oil Absorption Capacity (OAC), and Syneresis

The water absorption capacity (WAC) of AHT and OPT starches increased significantly compared to other treatments (Table 5). The increase in WAC was associated with the presence of the hydroxyl group and the loss of the starch crystalline structure. The physical modification reduced the starch crystalline structure and increased the tendency of starch to have hydrophilic properties, which increased the WAC [48]. The hydrophilic characteristic is caused by breaking the hydrogen bonds between the amorphous and crystalline regions, which expands the amorphous areas of the starch structure [49,50]. In addition, the increase in WAC in physically modified starch was also caused by changes in the structure of the starch granules, which were more porous so that it was easier to absorb water [18]. This result is in line with previous research on sago starch [21].

WAC between both chemical modifications, OSA and CA, was not significantly different, and was higher than native starch. The octenyl group penetrates the starch structure, which causes a change in the structural system of the starch by breaking the hydrogen bonds between the starch chains so that water penetration into the starch granules increases at low temperatures [51]. This result is in line with previous research on sago starch [52]. CA starch has many hydrophilic groups compared to native starch. The granule size of CA starch is smaller than native starch, which can increase WAC. The smaller the granule size, the larger the surface area of the starch granules, so that more water can penetrate the granule [53]. Furthermore, high acid concentrations increase the starch fraction with hydroxyl groups that can hold water molecules to form hydrogen bonds, thereby increasing WAC [54].

Oil absorption capacity (OAC) in AHT increased compared to the native starch (Table 5). The high OAC in AHT starch is associated with its granular morphology, which has a rough surface and forms cavities. Surface roughness and release of the helical structure of starch granules after hydrothermal modification increase the tendency of starch with lipophilic properties [55]. This result is in agreement with a previous study on hydrothermally modified millet starch [56]. OAC between OSA and CA starch was not significantly different, and decreased compared to the native starch. The decrease of OAC on CA starch is in line with previous research on taro starch [57].

Table 5 shows that all modifications of starch significantly decreased the syneresis of native sago starch. This means that both thermal and chemical modifications can increase the freeze–thaw stability of native sago. The most interesting thing is that both physical modifications can reduce the syneresis of native sago starch to 0%, which indicates that there is no water coming out of the gel.

The decrease in syneresis also occurred in taro starch modified using an autoclave [58]. The decrease in the percentage of syneresis can be caused by an increase in the amylose content of AHT- and OPT-modified starch so that a stronger amylose gel will resist the expulsion of water. In addition, the high heat originating from the autoclave also causes a rearrangement of the amylopectin molecules and results in the formation of more hydroxyl bonds, which can reduce the amount of water that comes out and reduce the syneresis value. There was also a significant decrease in the percentage of syneresis in OSA-modified starch and citric acid cross-linking. Song et al. [37] reported a decreased syneresis of modified corn starch and obtained a lower percentage of syneresis than native starch. This might be due to how, during the freezing process, hydrogen bonds are easily formed in the environment around the starch and cause the water between the starch molecules to be forced out of the gel so that the starch’s capacity to hold water decreases. However, the presence of COOH bonds in OSA-modified sago starch will retain water lost due to the addition of COOH, which causes hydration with water molecules to form intramolecular hydrogen bonds with dehydration of the starch chain hydroxy glucose [59]. A low percentage of syneresis was possessed by all physically and chemically modified sago starches, and this study proved that physical and chemical modifications increased the freeze–thaw stability of sago starch. It showed that these four modified starches are suitable as raw materials for frozen food products such as yogurt or other frozen foods.

## 4. Conclusions

Physical (autoclave-heating treatment and osmotic-pressure treatment) and chemical (octenyl-succinic anhydride and cross-linking citric acid) modifications had different and significant effects on the chemical composition, granule morphology, crystallinity, and functional properties of native sago starch. For chemical composition, modifications significantly affected the water content, but only autoclave-heating treatment (AHT) significantly affected amylose content. Whereas AHT and octenyl-succinic anhydride (OSA) modifications significantly affected starch content. Physical modifications damaged the starch granules, while chemical modifications only slightly damaged the granules. All modifications did not change the type of crystallinity through the crystallinity pattern but decreased the relative crystallinity. In addition, there was no change in functional groups in physically modified sago starch, but new functional groups were formed in chemically modified sago starch at a wavelength of 1700–1725 cm^−1^. The degree of order (DO) and degree of double helix (DD) of the four modified starches were also not significantly different from the native sago starch, except for AHT starch which decreased the DO value, and osmotic-pressure treatment (OPT), which increased the DD value. The functional properties of sago starch underwent different changes after the modification process. Freeze–thaw stability or the percentage of syneresis of sago starch decreased in all modified sago starch. Physical modification decreased the swelling volume value, while chemical modification increased its value, and this was inversely proportional to the solubility of all physically modified starch, which increased while chemical modification decreased. All modifications improved the water absorption capacity (WAC), while oil absorption capacity (OAC) with the physical modification increased and with the chemical modification decreased. All modified starches had high freeze–thaw stability (low syneresis), indicating that this starch has the potential to be applied in frozen food.

## Figures and Tables

**Table 1:** Chemical composition of native and various modified sago starches.

Treatment	Moisture Content (%)	Starch Content (%)	Amylose Content (%)
Native	11.15 ± 0.07 ^a^	85.06 ± 0.52 ^c^	25.09 ± 0.86 ^bc^
AHT	10.14 ± 0.15 ^b^	86.01 ± 0.40 ^b^	26.23 ± 1.00 ^a^
OPT	10.59 ± 0.6 ^b^	85.47 ± 0.83 ^bc^	25.97 ± 1.51 ^ab^
OSA	8.84 ± 0.53 ^c^	87.16 ± 0.05 ^a^	24.85 ± 0.67 ^c^
CA	6.41 ± 0.36 ^d^	85.79 ± 0.01 ^bc^	25.80 ± 0.78 ^abc^

Means marked with different letters are significantly different (*p* < 0.05). AHT = autoclave-heating treatment, OPT = osmotic-pressure treatment, OSA = octenyl-succinic anhydride, CA = citric acid cross-linking.

**Table 2:** Particle size distribution of native and various modified sago starches.

Treatment	Particle Size Distribution (μm)				
	<10%	<50%	<90%	Mean	Median
Native	19.39	30.11	41.82	30.33	30.11
AHT	17.01	35.62	100.60	46.69	35.62
OPT	17.88	32.53	52.65	36.12	32.53
OSA	20.75	30.61	41.25	30.65	30.61
CA	18.95	29.40	40.32	29.41	29.40

AHT = autoclave-heating treatment, OPT = osmotic-pressure treatment, OSA = octenyl-succinic anhydride, CA = citric acid cross-linking.

**Table 3:** The degree of order (DO) and degree of double helix (DD) of native and modified sago starches.

Treatment	1047/1022 Ratio (DO)	995/1022 Ratio (DD)
Native	0.659 ± 0.01 ^a^	1.235 ± 0.14 ^bc^
AHT	0.640 ± 0.00 ^b^	1.257 ± 0.01 ^ab^
OPT	0.657 ± 0.01 ^a^	1.259 ± 0.03 ^a^
OSA	0.647 ± 0.17 ^ab^	1.217 ± 0.01 ^c^
CA	0.659 ± 0.11 ^a^	1.235 ± 0.02 ^bc^

Means marked with different letters are significantly different (*p* < 0.05). AHT = autoclave-heating treatment, OPT = osmotic-pressure treatment, OSA = octenyl-succinic anhydride, CA = citric acid cross-linking.

**Table 4:** Swelling volume and solubility of native and modified sago starches.

Treatment	Swelling Volume (%)	Solubility (%)
Native	24.04 ± 1.26 ^c^	12.42 ± 1.23 ^c^
AHT	13.24 ± 0.77 ^d^	18.14 ± 0.62 ^a^
OPT	11.88 ± 0.43 ^e^	17.07 ± 0.37 ^b^
OSA	28.88 ± 1.65 ^a^	4.10 ± 0.57 ^e^
CA	26.52 ± 0.58 ^b^	11.20 ± 0.73 ^d^

Means marked with different letters are significantly different (*p* < 0.05). AHT = autoclave-heating treatment, OPT = osmotic-pressure treatment, OSA = octenyl-succinic anhydride, CA = citric acid cross-linking.

**Table 5:** Water absorption capacity (WAC), oil absorption capacity (AOC), and syneresis of native and modified sago starches.

Treatment	WAC (g/g db)	OAC (g/g db)	Syneresis (%)
Native	0.72 ± 0.04 ^a^	0.97 ± 0.06 ^b^	6.26 ± 0.55 ^a^
AHT	1.92 ± 0.07 ^d^	1.08 ± 0.11 ^c^	0.00
OPT	1.65 ± 0.11 ^c^	0.98 ± 0.05 ^bc^	0.00
OSA	1.01 ± 0.28 ^b^	0.83 ± 0.03 ^a^	1.54 ± 0.07 ^b^
CA	0.94 ± 0.06 ^b^	0.84 ± 0.05 ^a^	1.02 ± 0.21 ^c^

Means marked with different letters are significantly different (*p* < 0.05). AHT = autoclave-heating treatment, OPT = osmotic-pressure treatment, OSA = octenyl-succinic anhydride, CA = citric acid cross-linking.

## Data Availability

Not applicable.

## References

[1] Bujang K., Ehara H., Toyoda Y., Johnson D.V. (2018). Production, Purification, and Health Benefits of Sago Sugar. Sago Palm: Multiple Contributions to Food Security and Sustainable Livelihoods.

[2] Nordin N.A., Rahman N.A., Talip N., Yacob N. (2018). Citric Acid Cross-Linking of Carboxymethyl Sago Starch Based Hydrogel for Controlled Release Application. Macromol. Symp..

[3] Marta H., Cahyana Y., Djali M. (2021). Densely Packed-Matrices of Heat Moisture Treated-Starch Determine the Digestion Rate Constant as Revealed by Logarithm of Slope Plots. J. Food Sci. Technol..

[4] Zavareze E.d.R., Dias A.R.G. (2011). Impact of Heat-Moisture Treatment and Annealing in Starches: A Review. Carbohydr. Polym..

[5] Pukkahuta C., Shobsngob S., Varavinit S. (2007). Effect of Osmotic Pressure on Starch: New Method of Physical Modification of Starch. Starch-Stärke.

[6] Korma S.A., Kamal-Alahmad S.N., Ammar A.F., Zaaboul F., Zhang T. (2016). Chemically Modified Starch and Utilization in Food Stuffs. Int. J. Nutr. Food Sci..

[7] Altuna L., Herrera M.L., Foresti M.L. (2018). Synthesis and Characterization of Octenyl Succinic Anhydride Modified Starches for Food Applications. A Review of Recent Literature. Food Hydrocoll..

[8] Bajaj R., Singh N., Kaur A. (2019). Properties of Octenyl Succinic Anhydride (Osa) Modified Starches and Their Application in Low Fat Mayonnaise. Int. J. Biol. Macromol..

[9] Mason W.R., BeMiller J., Whistler R. (2009). Chapter 20—Starch Use in Foods. Starch.

[10] Kim J.Y., Lee Y.-K., Chang Y.H. (2017). Structure and Digestibility Properties of Resistant Rice Starch Cross-Linked with Citric Acid. Int. J. Food Prop..

[11] Ghanbarzadeh B., Almasi H., Entezami A.A. (2011). Improving the Barrier and Mechanical Properties of Corn Starch-Based Edible Films: Effect of Citric Acid and Carboxymethyl Cellulose. Ind. Crops Prod..

[12] Marta H., Cahyana Y., Arifin H.R., Khairani L. (2019). Comparing the Effect of Four Different Thermal Modifications on Physicochemical and Pasting Properties of Breadfruit (*Artocarpus altilis*) Starch. Int. Food Res. J..

[13] Anwar S.H., Hasni D., Rohaya S., Antasari M., Winarti C. (2020). The Role of Breadfruit Osa Starch and Surfactant in Stabilizing High-Oil-Load Emulsions Using High-Pressure Homogenization and Low-Frequency Ultrasonication. Heliyon.

[14] Li J., Han W., Zhang B., Zhao S., Du H. (2018). Structure and Physicochemical Properties of Resistant Starch Prepared by Autoclaving-Microwave. Starch-Stärke.

[15] AOAC (1995). Official Method of Analysis.

[16] AOAC (1970). Official Method of Analysis.

[17] Graham R. (2002). A Proposal for Irri to Establish a Grain Quality and Nutrition Research Center.

[18] Marta H., Cahyana Y., Bintang S., Soeherman G.P., Djali M. (2022). Physicochemical and Pasting Properties of Corn Starch as Affected by Hydrothermal Modification by Various Methods. Int. J. Food Prop..

[19] Marta H., Cahyana Y., Djali M., Arcot J., Tensiska T. (2019). A Comparative Study on the Physicochemical and Pasting Properties of Starch and Flour from Different Banana (*Musa* spp.) Cultivars Grown in Indonesia. Int. J. Food Prop..

[20] Marta H., Cahyana Y., Djali M. (2021). Pectin Interaction with Thermally Modified Starch Affects Physicochemical Properties and Digestibility of Starch as Revealed by Logarithm of Slop Plot. CyTA J. Food.

[21] Dewi A.M., Santoso U., Pranoto Y., Marseno D.W. (2022). Dual Modification of Sago Starch Via Heat Moisture Treatment and Octenyl Succinylation to Improve Starch Hydrophobicity. Polymers.

[22] Wattanachant S., Muhammad K., Mat Hashim D., Rahman R.A. (2003). Effect of Crosslinking Reagents and Hydroxypropylation Levels on Dual-Modified Sago Starch Properties. Food Chem..

[23] Remya R., Jyothi A.N., Sreekumar J. (2018). Effect of Chemical Modification with Citric Acid on the Physicochemical Properties and Resistant Starch Formation in Different Starches. Carbohydr. Polym..

[24] Uthumporn U., Wahidah N., Karim A.A. (2014). Physicochemical Properties of Starch from Sago (*Metroxylon sagu*) Palm Grown in Mineral Soil at Different Growth Stages. IOP Conf. Ser. Mater. Sci. Eng..

[25] Karmakar R., Ban D., Ghosh U. (2014). Comparative Study of Native and Modified Starches Isolated from Conventional and Nonconventional Sources. Int. Food Res. J..

[26] Lopez-Silva M., Bello-Perez L.A., Agama-Acevedo E., Alvarez-Ramirez J. (2019). Effect of Amylose Content in Morphological, Functional and Emulsification Properties of Osa Modified Corn Starch. Food Hydrocoll..

[27] Zainal Abiddin N.F., Yusoff A., Ahmad N. (2018). Effect of Octenylsuccinylation on Physicochemical, Thermal, Morphological and Stability of Octenyl Succinic Anhydride (Osa) Modified Sago Starch. Food Hydrocoll..

[28] Guo J., Tang W., Quek S.Y., Liu Z., Lu S., Tu K. (2020). Evaluation of Structural and Physicochemical Properties of Octenyl Succinic Anhydride Modified Sweet Potato Starch with Different Degrees of Substitution. J. Food Sci..

[29] Xu J., Zhou C.-w., Wang R.-z., Yang L., Du S.-s., Wang F.-p., Ruan H., He G.-q. (2012). Lipase-Coupling Esterification of Starch with Octenyl Succinic Anhydride. Carbohydr. Polym..

[30] Liang S., Hong Y., Gu Z., Cheng L., Li C., Li Z. (2021). Effect of Debranching on the Structure and Digestibility of Octenyl Succinic Anhydride Starch Nanoparticles. LWT-Food Sci. Technol..

[31] Zhou J., Tong J., Su X., Ren L. (2016). Hydrophobic Starch Nanocrystals Preparations through Crosslinking Modification Using Citric Acid. Int. J. Biol. Macromol..

[32] No J., Mun S., Shin M. (2019). Properties and Digestibility of Octenyl Succinic Anhydride-Modified Japonica-Type Waxy and Non-Waxy Rice Starches. Molecules.

[33] Chen M., Yin T., Chen Y., Xiong S., Zhao S. (2014). Preparation and Characterization of Octenyl Succinic Anhydride Modified Waxy Rice Starch by Dry Media Milling. Starch-Stärke.

[34] Whitney K., Reuhs B.L., Ovando Martinez M., Simsek S. (2016). Analysis of Octenylsuccinate Rice and Tapioca Starches: Distribution of Octenylsuccinic Anhydride Groups in Starch Granules. Food Chem..

[35] Hui R., Qi-he C., Ming-liang F., Qiong X., Guo-qing H. (2009). Preparation and Properties of Octenyl Succinic Anhydride Modified Potato Starch. Food Chem..

[36] Rashid I., Omari M.H.A., Leharne S.A., Chowdhry B.Z., Badwan A. (2012). Starch Gelatinization Using Sodium Silicate: FTIR, DSC, XRPD, and NMR Studies. Starch-Stärke.

[37] Song X., Zhu W., Li Z., Zhu J. (2010). Characteristics and Application of Octenyl Succinic Anhydride Modified Waxy Corn Starch in Sausage. Starch-Stärke.

[38] Zhang B., Mei J.-Q., Chen B., Chen H.-Q. (2017). Digestibility, Physicochemical and Structural Properties of Octenyl Succinic Anhydride-Modified Cassava Starches with Different Degree of Substitution. Food Chem..

[39] Lipatova I.M., Yusova A.A. (2021). Effect of Mechanical Activation on Starch Crosslinking with Citric Acid. Int. J. Biol. Macromol..

[40] Reddy N., Yang Y. (2010). Citric Acid Cross-Linking of Starch Films. Food Chem..

[41] Velásquez-Barreto F.F., Bello-Pérez L.A., Yee-Madeira H., Alvarez-Ramirez J., Velezmoro-Sánchez C.E. (2020). Effect of the Osa Esterification of Oxalis Tuberosa Starch on the Physicochemical, Molecular, and Emulsification Properties. Starch-Stärke.

[42] Zheng M.-z., Xiao Y., Yang S., Liu H.-m., Liu M.-h., Yaqoob S., Xu X.-y., Liu J.-s. (2020). Effects of Heat–Moisture, Autoclaving, and Microwave Treatments on Physicochemical Properties of Proso Millet Starch. Food. Sci. Nutr..

[43] Bhosale R., Singhal R. (2007). Effect of Octenylsuccinylation on Physicochemical and Functional Properties of Waxy Maize and Amaranth Starches. Carbohydr. Polym..

[44] Gayary M.A., Mahanta C.L. (2020). Optimization of Process Parameters of Osmotic Pressure Treatment and Heat Moisture Treatment for Rice Starch Using Response Surface Methodology. J. Food Meas. Charact..

[45] Pukkahuta C., Suwannawat B., Shobsngob S., Varavinit S. (2008). Comparative Study of Pasting and Thermal Transition Characteristics of Osmotic Pressure and Heat–Moisture Treated Corn Starch. Carbohydr. Polym..

[46] Utomo P., Nizardo N.M., Saepudin E. (2020). Crosslink Modification of Tapioca Starch with Citric Acid as a Functional Food. AIP Conference Proceedings.

[47] Xiao H.X., Lin Q.L., Liu G.Q., Yu F.X. (2012). A Comparative Study of the Characteristics of Cross-Linked, Oxidized and Dual-Modified Rice Starches. Molecules.

[48] Bharti I., Singh S., Saxena D.C. (2019). Exploring the Influence of Heat Moisture Treatment on Physicochemical, Pasting, Structural and Morphological Properties of Mango Kernel Starches from Indian Cultivars. LWT -Food Sci. Technol..

[49] Sindhu R., Khatkar B.S. (2018). Thermal, Structural and Textural Properties of Amaranth and Buckwheat Starches. J. Food Sci. Technol..

[50] Solaesa Á.G., Villanueva M., Muñoz J.M., Ronda F. (2021). Dry-Heat Treatment vs. Heat-Moisture Treatment Assisted by Microwave Radiation: Techno-Functional and Rheological Modifications of Rice Flour. LWT-Food Sci. Technol..

[51] Sharma M., Singh A.K., Yadav D.N., Arora S., Vishwakarma R.K. (2016). Impact of Octenyl Succinylation on Rheological, Pasting, Thermal and Physicochemical Properties of Pearl Millet (*Pennisetum typhoides*) Starch. LWT -Food Sci. Technol..

[52] Naseri A., Shekarchizadeh H., Kadivar M. (2019). Octenylsuccination of Sago Starch and Investigation of the Effect of Calcium Chloride and Ferulic Acid on Physicochemical and Functional Properties of the Modified Starch Film. J. Food Process. Preserv..

[53] BeMiller J.N., Huber K.C. (2015). Physical Modification of Food Starch Functionalities. Annu. Rev. Food Sci. Technol..

[54] Babu A.S., Parimalavalli R., Jagannadham K., Rao J.S. (2015). Chemical and Structural Properties of Sweet Potato Starch Treated with Organic and Inorganic Acid. J. Food Sci. Technol..

[55] Wang Q., Li L., Zheng X. (2021). Recent Advances in Heat-Moisture Modified Cereal Starch: Structure, Functionality and Its Applications in Starchy Food Systems. Food Chem..

[56] Sharma M., Yadav D., Singh A.K., Tomar S. (2015). Effect of Heat-Moisture Treatment on Resistant Starch Content as Well as Heat and Shear Stability of Pearl Millet Starch. Agric. Res..

[57] Falade K.O., Ayetigbo O.E. (2015). Effects of Annealing, Acid Hydrolysis and Citric Acid Modifications on Physical and Functional Properties of Starches from Four Yam (*Dioscorea* spp.) Cultivars. Food Hydrocoll..

[58] Deka D., Sit N. (2016). Dual Modification of Taro Starch by Microwave and Other Heat Moisture Treatments. Int. J. Biol. Macromol..

[59] Wang J., Su L., Wang S. (2010). Physicochemical Properties of Octenyl Succinic Anhydride-Modified Potato Starch with Different Degrees of Substitution. J. Sci. Food Agric..

